# A Taxonomic Revision of the East Mediterranean Species of the *Crematogaster scutellaris* Complex (Hymenoptera: Formicidae)

**DOI:** 10.3390/insects17060658

**Published:** 2026-06-22

**Authors:** Sándor Csősz, Laura El-Ghor, Herbert C. Wagner

**Affiliations:** 1 HUN-REN-ELTE-MTM Integrative Ecology Research Group, Pázmány Péter ave 1/C, 1117 Budapest, Hungary; sandorcsosz2@gmail.com; 2 Department of Systematic Zoology and Ecology, Institute of Biology, ELTE-Eötvös Loránd University, Pázmány Péter ave 1/C, 1117 Budapest, Hungary; eglaura@student.elte.hu; 3 Institute of Biology, University of Graz, Universitätsplatz 2/I, 8010 Graz, Austria

**Keywords:** *Crematogaster ionia* Forel, 1911, Crete, cryptic species, Greece, morphometrics, nest-centroid clustering

## Abstract

The taxonomy of the East Mediterranean species of the *Crematogaster scutellaris* complex, *Crematogaster schmidti* (Mayr, 1853) and *C. ionia* Forel, 1911 sensu lato, has not yet been investigated via modern approaches like morphometric analyses. We collected morphometric data of 201 workers from 68 nests of *Crematogaster schmidti* and *C. ionia* s. l. from Slovenia, Croatia, Montenegro, North Macedonia, the Greek mainland, Crete, Bulgaria, Samos, Karpathos, Rhodes, Turkish Thrace, and Anatolia. Nest-centroid clustering suggested four distinct entities with different geographic distributions: *C. schmidti* from Slovenia southwards to Greece and Turkish Thrace, and three species which have been so far summarized under *C. ionia*: one from the Greek mainland and North Macedonia, one from Crete, and one from Samos, Karpathos, Rhodes, and Anatolia. We describe two new species: the Cretan entity as *Crematogaster ariadnae* sp. n. and the Balkan mainland entity as *Crematogaster graeca* sp. n. A key and (re)descriptions for the East Mediterranean members of the *Crematogaster scutellaris* complex are provided. The four species show different geographic distribution patterns, do not occur together at the same localities, and most likely speciated through long-term geographic isolation.

## Simple Summary

Ant species of the Mediterranean *Crematogaster scutellaris* complex are very common and ecologically important, but they are extremely difficult to distinguish because of their very similar appearance. In this study, we investigated 201 ant individuals from 68 nests collected on the Balkan mainland, Aegean islands, and Anatolia using detailed body measurements. Our aim was to clarify species boundaries and to test whether hidden, previously unrecognized species exist within this complex. We recorded 15 body measurements for each individual and analyzed them using statistical methods. The results revealed four clearly distinct morphological entities which we consider to be biological species. Two of them, *Crematogaster graeca* sp. n. and *Crematogaster ariadnae* sp. n., are described here as new to science. We also provide identification characters that allow for the reliable separation of these species. Our findings improve understanding of Mediterranean ant diversity and provide a basis for future ecological and biogeographic research.

## 1. Introduction

Worldwide, there are currently 530 valid species and 258 subspecies of the genus *Crematogaster*, divided into the two subgenera *Crematogaster* and *Orthocrema* [1]. The 8 million-year-old *Crematogaster scutellaris* group forms a distinct clade within the subgenus *Crematogaster* and is mainly distributed in the Holarctic [2,3]. In the New World alone, 37 species have been listed [3], but the number of species occurring in the Palearctic is unknown. Within the *C. scutellaris* group [2], we define the West Palearctic species which are obviously closely related with *C. scutellaris* (Olivier, 1792) as the *C. scutellaris* complex. They diverged approximately 4 million years ago [3,4] and can be separated from other Mediterranean *Crematogaster* species by their long spines [5,6], the reduced pilosity of their first gastral tergite [7,8], and the distinct rugae on their pronotum [8]. All species of the *C. scutellaris* complex nest in dead wood, while species outside the complex native to Europe—for example, *Crematogaster auberti* Emery, 1869—nest in soil or under stones [6,7,9,10].

Species of the *Crematogaster scutellaris* complex are among the most ecologically dominant ants of Mediterranean woodland biotopes [11,12,13,14]. They occur in light xerothermous broadleaf and coniferous forests as well as on single trees and they build large carton nests in cavities of trees, dead logs, and under bark [6,9,10,14,15,16,17,18,19]. Colonies have been estimated to contain several thousand to several 10,000 workers [15,17,19,20,21]; they form densely trafficked foraging trails from their nests to aphid colonies [19,22,23,24]. Trails are marked with tridecan-2-ol [25] secreted from tibial glands [26] and are commonly followed also by Camponotini ants [22,23,24,27,28,29,30]. Workers are highly territorial and aggressive [17,30,31,32]. They put up their gaster in a scorpion-like manner and defend themselves by secreting contact poisons [6,17,32,33,34,35] which make them unpalatable to vertebrate predators [30,36]. Some species exhibit conspicuous colors acting as aposematic signals [37,38,39].

Currently, three described taxa of this complex within the study area are recognized as valid species: *Crematogaster scutellaris*, described from France [40], is a West-Mediterranean species occurring in Northwest Africa and Iberia, on the Balearic Islands, in Southern France and mainland Italy, on Sardinia, Corsica, and Sicily, in Istria, on most Croatian islands, and along the Dalmatian mainland coast southwards until the latitude of Island Hvar [38]. This species typically has a reddish head while the rest of the body is blackish, and workers and gynes are larger than in the other species of the complex [17,37]. *Crematogaster schmidti* (Mayr, 1853), described from Slovenia [41], occurs in Northeast Italy [42,43], Slovenia [18,41], Northern Istria, Hungary [44], the Central Balkans [45,46,47], and Dalmatia south of the latitude of the Island Hvar [48,49], southwards to Greece [50,51]. It also occurs in Anatolia [52,53], Crimea [21], in the Caucasus [54], and eastwards until Iran [55]. Typically, *C. schmidti* workers differ from those of *C. scutellaris* by having a reddish mesosoma and a reddish waist; species delimitation by pigmentation is believed to be stronger than typically known in ants [17,37]. A morphometric differentiation between *C. scutellaris* and *C. schmidti* is possible [17]. *Crematogaster ionia* Forel, 1911 sensu lato, described from Greece and Anatolia [56], is a species with typically homogeneously dark brownish coloration [7,37,56] which occurs in the southernmost regions of the Balkan mainland, on most or all Greek islands, in the southwestern half of Anatolia, in Cyprus, and along the Mediterranean to the southeast until Israel [38].

While the morphometric delimitation of *C. scutellaris* from *C. schmidti* is well-justified and introgression between them has been investigated [17,18], no morphometric key to distinguish *C. ionia* from *C. schmidti* is available. Instead, subjective color assessment seems to be still the state-of-the-art method to distinguish the homogeneously dark brownish *C. ionia* s. l. from the bicolored *C. schmidti* [7,8,30,48,57,58]. In addition to the unclear delimitation from *C. schmidti*, the taxonomy within *C. ionia* s. l. also remains unresolved. The possibility of additional species within the complex has been suggested several times [7,58,59,60]; such putative undescribed species are often pragmatically summarized under *C. ionia* [37,38].

This study presents the delimitation of *C. schmidti*, *C. ionia* s. str., and two further entities previously summarized under *C. ionia* and *C. schmidti*. We newly describe the species endemic to Crete as *Crematogaster ariadnae* sp. n. and the species occurring in the Greek mainland and North Macedonia as *Crematogaster graeca* sp. n.

## 2. Materials and Methods

### 2.1. Material Examination

#### 2.1.1. Material

The investigated ant material was mainly collected by Herbert C. Wagner (HCW) in 2010–2024. This revision considers the two most similar species, *C. schmidti* and *C. ionia*, for delimitation, but excludes *C. scutellaris*, which is, for zoogeographic reasons, not considered to be potentially confused or synonymous with *C. ionia* s. l. We measured 15 continuous morphometric traits in 201 workers from 68 nest samples in the East Mediterranean Basin, with a focus on the Aegean. The complete list of examined materials is provided in Supplementary Table S1. Specimens are deposited in the following institutions: Muséum d’histoire naturelle, Geneva, Switzerland (MHNG); the Zoological Museum of the Humboldt University Berlin (ZMB), Germany (MNHB); Naturhistorisches Museum Wien, Austria (NHMW); Senckenberg Museum für Naturkunde Görlitz, Germany (SMNG), the private collection of SC (SCPC), and the private collection of HCW (HCWPC). The type series of taxa considered within this revisionary work were investigated by direct morphometric investigation or through the AntWeb.org online database. In the latter cases, images were taken via tpsDig2 Version 2.32 freeware to measure the standard morphometric characters (Table S1). To enhance the accuracy and reliability of the morphometric results, the recommendations of Csősz et al. [61] were considered.

#### 2.1.2. Protocol for Morphometric Character Recording

**Figure 1:**
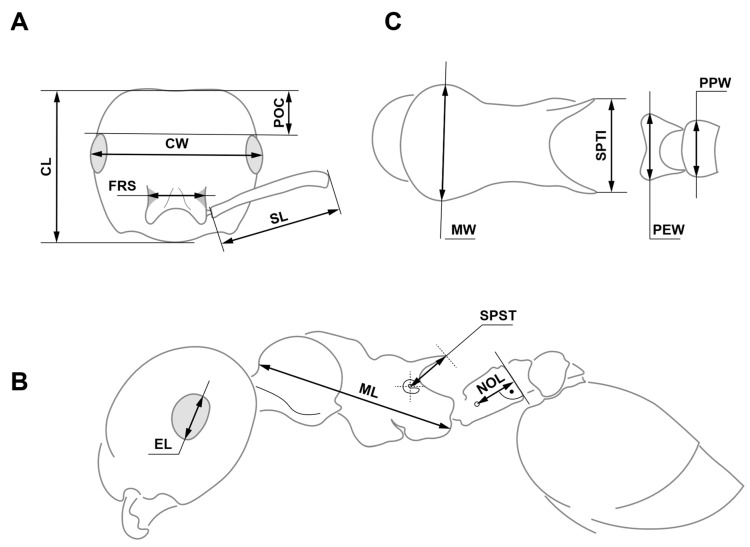
Illustrations for measured morphometric characters. (**A**): full-face view; (**B**): dorsal view of mesosoma and waist; (**C**): lateral view of a *Crematogaster* worker.

All measurements were recorded in µm using a pin-holding stage that allows rotation around the *X*, *Y*, and *Z* axes. An Olympus SZX16 stereomicroscope (Olympus Corporation, Tokyo, Japan) at ×120 magnification was employed for all character measurements. Morphometric data are presented in µm throughout this paper. The shape variables of most specimens were measured by Laura El-Ghor, and the pilosity parameters mainly by Sándor Csősz. Only workers were included in the analysis. Detailed definitions of morphometric characters are presented in Table 1 and Figure 1A–C.

### 2.2. Multivariate Statistics—Establishing the Morphospecies Hypothesis

#### 2.2.1. Exploratory Analyses Using NC-PART Clustering

The preliminary species hypothesis was established based on worker morphometrics using a combination of NC clustering, UPGMA distance method [62], and Partitioning-Based Recursive Thresholding (PART) [63]. We followed a published protocol [64] with the following parameters: bootstrap iterations in PART were set to ‘b = 1000’, and the minimum cluster size was set to ‘minSize = 5’ for ‘hclust’. Since linear discriminant analyses (LDA) yield more robust results than NC clustering, samples with doubtful classification in the NC cluster, as well as type workers, were evaluated as wild cards in an LDA [62]. Finally, overall accuracy on the worker and nest level was evaluated in a full-data LDA; nest-level classification probabilities were calculated as the geometric means of the classification probabilities of workers from the same nest.

#### 2.2.2. Allometric Correction for Principal Component Analyses

An alternative prior species hypothesis has been generated via principal component analyses (PCAs) of allometrically corrected data. Allometries (i.e., disproportionate body ratios with increasing body size [65]) can obscure morphological differences among biologically meaningful entities, because ant body size is strongly influenced by environmental conditions during ontogenesis, particularly larval nutrition [66,67]. Therefore, we removed the allometric effect by regressing each trait against CL as the independent variable (Table 1) and using the residual-corrected trait values for PCAs. PCAs are variance-driven and often strongly influenced by allometric size variation; consequently, allometric correction typically improves the separation of species by reducing size-dependent covariance and emphasizing shape-related differences among taxa [68,69,70].

In contrast, the removal of allometry was not necessary for NC clustering, which first transforms the data into a discriminant space and then plots the resulting discriminant values on a hierarchical cluster [38,62,64,71]. Since the first step operates on an LDA basis, normalization or allometric correction is unnecessary, as LDA itself is not sensitive to allometric relationships.

#### 2.2.3. Hypothesis Testing Through Confirmatory Analyses

We tested the validity of the preliminary morphospecies hypothesis using an LDA, placing the distributions of individuals in morphospace. A leave-one-out cross-validation LDA (LOOCV-LDA) provided a more conservative estimate of the model’s validity by iteratively testing each data point’s predicted position when it is excluded from the model. We tested each species pair separately by excluding all individuals of the two other species from the analyses. For each pair, two to eight characters were used. To avoid overfitting, the number of individuals of the smallest group had to be at least three times higher than the number of characters used [72].

### 2.3. Visual Analysis and Geographic Distribution

The distribution of one morphometric parameter is shown as a boxplot generated with the ggplot2 and gridExtra packages [73]. Distribution maps for the studied samples were generated using QGIS 3.32 [74].

## 3. Results

### 3.1. Morphological Pattern Recognition

**Figure 2:**
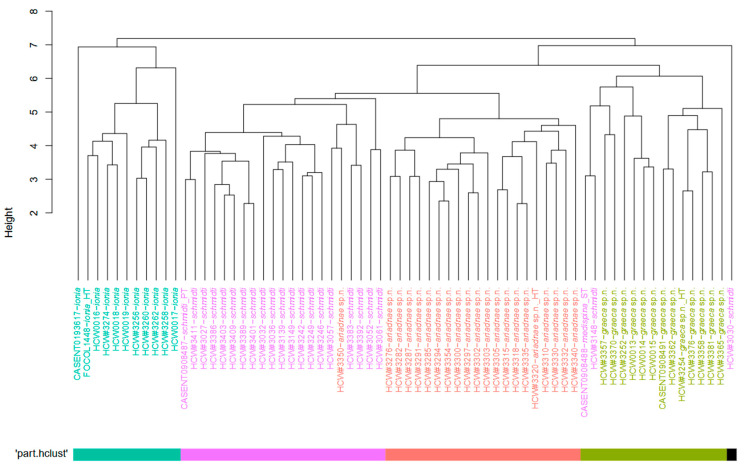
Dendrogram solution for morphometric data of *Crematogaster scutellaris* complex species in NC clustering, using UPGMA distance method. Pattern is calculated from raw data, and labels represent nest samples. Bars represent ‘hclust’ partitioning results returned by PART function. Colors of nest-sample labels show final species hypothesis.

Unsupervised multivariate analyses of morphometric data—NC-clustering combined with the partitioning PART algorithm (‘hclust’)—provided four distinct clusters (Figure 2). These morphologically cohesive units corresponded to biological species: *Crematogaster ariadnae* sp. n.; *C. graeca* sp. n.; *C. ionia* Forel, 1911; and *C. schmidti* (Mayr, 1853).

A full-data confirmatory LDA of the four-cluster morphological species hypothesis yielded an overall accuracy of 0.93 on the worker level and of 1.0 on the nest-mean level when all morphometric characters were included (14 out of 201 workers and none of the 68 nests were misclassified). 

The six investigated type workers were assigned to their respective species with classification probabilities of 0.620–0.990 (Table 2). While three types had values > 0.9, the *C. ionia* syntype FOCOL1448 had 0.711 for *C. ionia*, 0.148 for *C. schmidti*, and 0.130 for *C. graeca* sp. n. We consider the conspecificity with *C. schmidti* or *C. graeca* sp. n. as unlikely because of their homogeneous dark color and zoogeography. The probability values of *C. christowitchii* Forel, 1892 syntypes were < 0.9 for assignment to *C. schmidti*, while the second highest values were for *C. ariadnae* sp. n.; we exclude conspecificity with the latter due to coloration and zoogeography.

**Figure 3:**
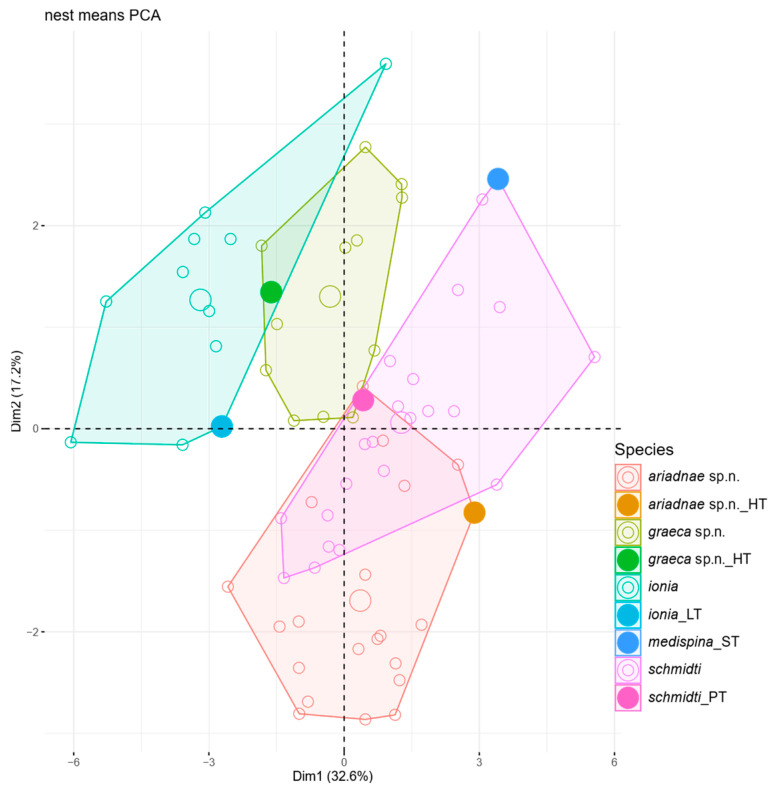
Nest-sample means of workers of the *Crematogaster scutellaris* complex species plotted on the principal component axes (PC1 and PC2). All allometrically corrected morphometric characters, with CL as the independent variable, were used. Large symbols represent species means. The positions of type workers are marked.

**Figure 4:**
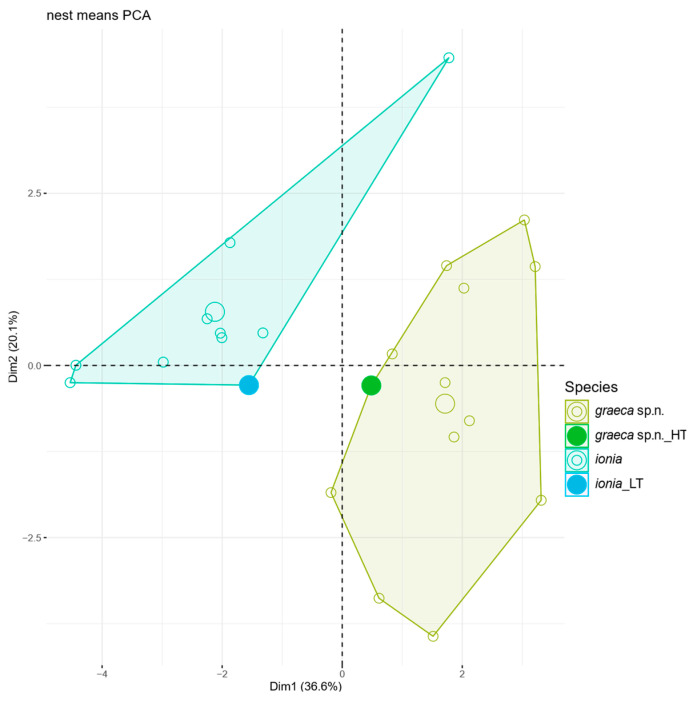
Nest-sample means of workers of *Crematogaster graeca* sp. n. and *C. ionia* plotted on the principal component axes (PC1 and PC2). All allometrically corrected morphometric characters, with CL as the independent variable, were used. The large symbols represent species means. The positions of type workers are marked.

**Figure 5:**
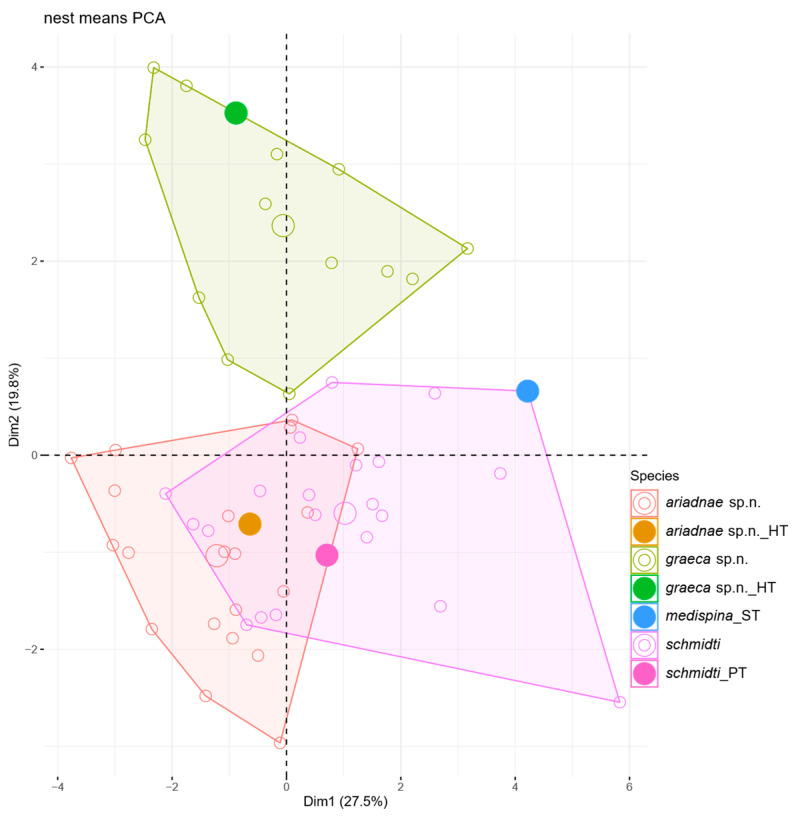
Nest-sample means of workers of the *Crematogaster scutellaris* complex, under the exclusion of *C. ionia*, plotted on the principal component axes (PC1 and PC2). All allometrically corrected morphometric characters, with CL as the independent variable, were used. The large symbols represent species means. The positions of type workers are marked.

**Figure 6:**
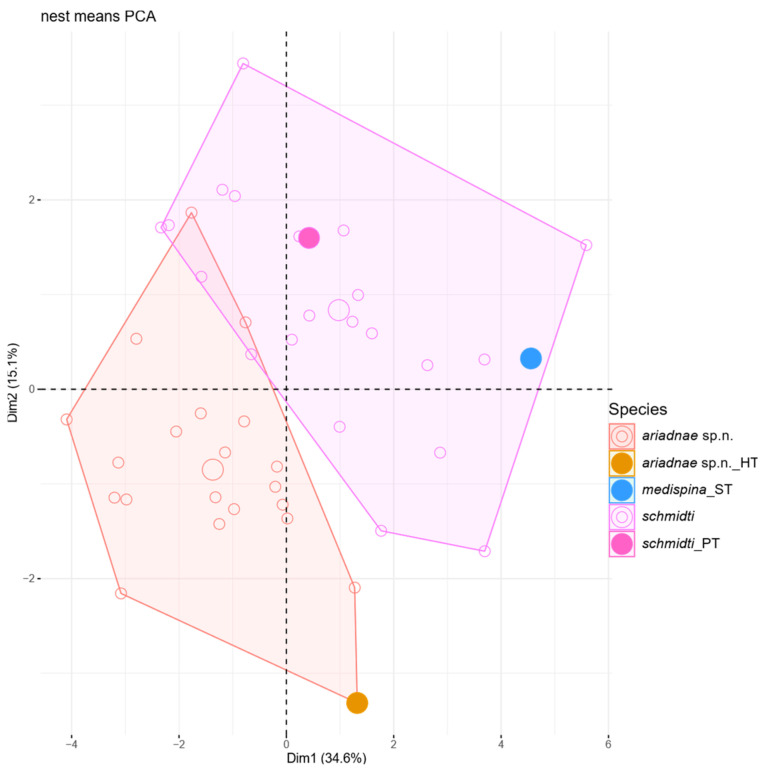
Nest-sample means of workers of the two morphologically closely related *Crematogaster scutellaris* complex species, *C. ariadnae* sp. n. and *C. schmidti*, plotted on the principal component axes (PC1 and PC2). All allometrically corrected morphometric characters, with CL as the independent variable, were used. The large symbols represent species means. The positions of type workers are marked.

PCAs based on nest means and all characters resulted in partial separation between species. In detail, there were small overlaps between *C. graeca* sp. n. and *C. ionia* as well as between *C. graeca* and *C. ariadnae*/*C. schmidti*, while *C. schmidti* largely overlapped with *C. ariadnae* sp. n. (Figure 3). A further PCA with only *C. graeca* sp. n. and *C. ionia* showed a clear separation between these two species (Figure 4). Also, a PCA with all samples excluding *C. ionia* showed a clear separation of *C. graeca* sp. n. from *C. ariadnae* sp. n. and *C. schmidti* (Figure 5). Finally, a PCA with only the latter two taxa showed slight overlap between these very similar species, with two *C. ariadnae* sp. n. samples falling within the polygon of *C. schmidti* (Figure 6). This limitation of the unsupervised method PCA was addressed for practical identification by discriminant analyses, which correctly classified 100% of nest means in our dataset (see Section 3.4 and Section 3.5).

Although the separation of *C. ionia* and *C. schmidti* has traditionally relied heavily on coloration [8,10,48,60], our analyses suggest that intermediate colorations occur; therefore, our identification key and diagnoses rely primarily on combinations of linear morphometric characters.

### 3.2. Confirmatory Analyses

We set a four-cluster morphological species hypothesis that was tested via confirmatory LOOCV-LDAs. Six combinations for pairwise species comparisons were available. The process returned LOOCV error rates between 0.0% and 5.8%, with the highest value observed for the pair *C. graeca* sp. n. and *C. ionia* (Table 3). As the confirmatory tests of the four-species hypothesis derived from NC clustering yielded a mean LOOCV error rate of 2.6%, we accepted the delimitation of four species as the final species hypothesis. This meets the criterion of the ‘Gene and Gene Expression species concept’ [75].

Of CS and 14 morphometric indices (Table 4), 5 to 11 traits were significantly different between species pairs, whereas *C. schmidti* and *C. ionia* showed the highest number of differences from each other and *C. schmidti* and *C. ariadnae* sp. n. the fewest (Table 5).

### 3.3. Geographic Distribution

**Figure 7:**
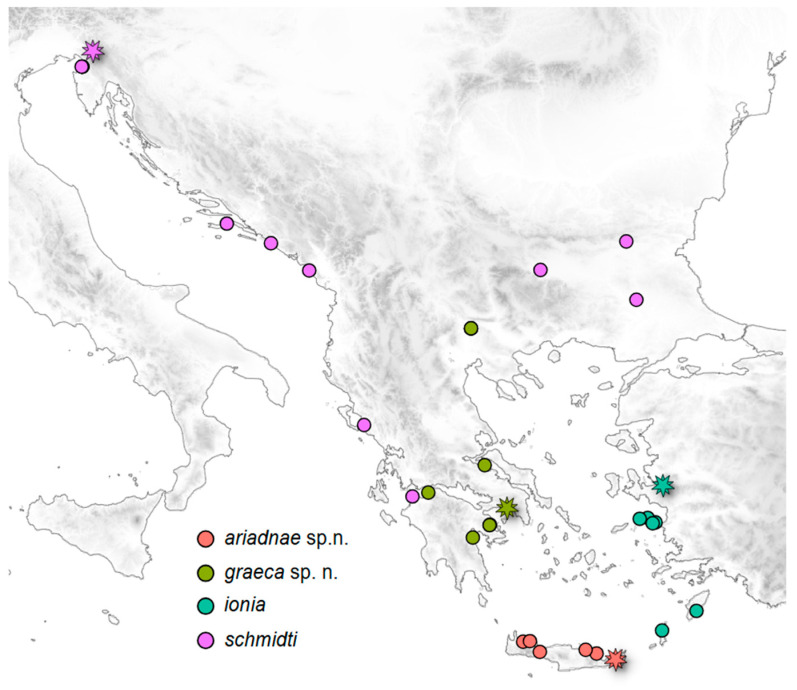
Localities of *Crematogaster* material investigated in this study. Type localities are shown as stars.

We found *Crematogaster ariadnae* sp. n. at six localities on Crete, *C. graeca* sp. n. at eight localities in Greece and North Macedonia, *C. ionia* at seven localities on the Aegean Islands (Karpathos, Samos, and Rhodes) and in Western Anatolia, and *C. schmidti* at eleven localities on the Balkans (Figure 7). We never found two species in the same locality.

**Table 4:** The mean of CS and morphometric indices calculated for *Crematogaster ariadnae* sp. n., *C. graeca* sp. n., *C. ionia*, and *C. schmidti* based on individuals (raw data) in μm. The upper row in fields gives arithmetic mean ± 95% confidence interval of the species mean; the lower row, in square brackets, the lower and upper extremes.

Character	*C. ariadnae* sp. n. (*n* = 66)	*C. graeca* sp. n. (*n* = 40)	*C. ionia* (*n* = 29)	*C. schmidti* (*n* = 66)
CS	971 ± 14 [869, 1127]	1034 ± 17 [942, 1160]	993 ± 18 [891, 1071]	974 ± 15 [816, 1136]
CL/CW	0.905 ± 0.005 [0.843, 0.959]	0.898 ± 0.006 [0.864, 0.963]	0.900 ± 0.004 [0.884, 0.923]	0.902 ± 0.004 [0.871, 0.932]
POC/CL	0.295 ± 0.002 [0.278, 0.316]	0.287 ± 0.003 [0.268, 0.310]	0.301 ± 0.004 [0.280, 0.324]	0.298 ± 0.003 [0.280, 0.336]
FRS/CS	0.388 ± 0.003 [0.362, 0.415]	0.380 ± 0.003 [0.360, 0.396]	0.366 ± 0.003 [0.347, 0.387]	0.387 ± 0.003 [0.364, 0.435]
SL/CS	0.787 ± 0.004 [0.758, 0.821]	0.800 ± 0.005 [0.771, 0.845]	0.788 ± 0.006 [0.759, 0.813]	0.784 ± 0.004 [0.750, 0.837]
MW/CS	0.618 ± 0.003 [0.579, 0.653]	0.622 ± 0.005 [0.594, 0.651]	0.610 ± 0.008 [0.572, 0.676]	0.624 ± 0.003 [0.595, 0.663]
SPTI/CS	0.461 ± 0.007 [0.358, 0.528]	0.467 ± 0.009 [0.416, 0.531]	0.432 ± 0.011 [0.376, 0.482]	0.478 ± 0.007 [0.426, 0.554]
PEW/CS	0.365 ± 0.004 [0.316, 0.400]	0.355 ± 0.005 [0.329, 0.384]	0.350 ± 0.006 [0.319, 0.389]	0.374 ± 0.005 [0.345, 0.430]
PPW/CS	0.299 ± 0.003 [0.269, 0.342]	0.296 ± 0.004 [0.269, 0.320]	0.291 ± 0.004 [0.273, 0.317]	0.310 ± 0.003 [0.277, 0.329]
ML/CS	1.087 ± 0.006 [1.020, 1.142]	1.098 ± 0.007 [1.058, 1.147]	1.059 ± 0.008 [1.010, 1.104]	1.083 ± 0.006 [1.029, 1.157]
SPST/CS	0.213 ± 0.003 [0.187, 0.257]	0.217 ± 0.005 [0.182, 0.244]	0.205 ± 0.005 [0.176, 0.241]	0.221 ± 0.003 [0.197, 0.251]
NOL/CS	0.250 ± 0.003 [0.214, 0.280]	0.243 ± 0.003 [0.222, 0.264]	0.236 ± 0.004 [0.219, 0.253]	0.249 ± 0.003 [0.214, 0.272]
EL/CS	0.253 ± 0.002 [0.234, 0.266]	0.255 ± 0.003 [0.235, 0.273]	0.247 ± 0.002 [0.236, 0.258]	0.254 ± 0.002 [0.241, 0.271]
PLG/CS	0.054 ± 0.001 [0.046, 0.064]	0.063 ± 0.002 [0.050, 0.074]	0.070 ± 0.002 [0.062, 0.079]	0.063 ± 0.001 [0.055, 0.073]
GHL/CS	0.088 ± 0.002 [0.072, 0.113]	0.100 ± 0.003 [0.087, 0.126]	0.105 ± 0.003 [0.087, 0.121]	0.100 ± 0.001 [0.090, 0.112]

**Table 5:** Characters with significant differences (*t*-test, 2 sides, type 2) between two species after Bonferroni–Holm correction [76] marked with checkmarks.

	CS	CL/CW	POC/CL	FRS/CS	SL/CS	MW/CS	SPTI/CS	PEW/CS	PPW/CS	ML/CS	SPST/CS	NOL/CS	EL/CS	PLG/CS	GHL/CS
***ariadnae*-*graeca***	✓		✓	✓	✓									✓	✓
***ariadnae*-*ionia***				✓			✓	✓		✓		✓	✓	✓	✓
***ariadnae*-*schmidti***							✓		✓		✓			✓	✓
***graeca-ionia***			✓	✓			✓			✓			✓	✓	
***graeca-schmidti***	✓		✓	✓	✓			✓	✓						
***ionia-schmidti***				✓		✓	✓	✓	✓	✓	✓	✓	✓	✓	✓

### 3.4. Key to Workers of the East Mediterranean Species Crematogaster scutellaris Complex

**1.**  Pubescence and hairs on 1^st^ gastral tergite shorter: PLG/CS = 0.046–0.064, GHL/CS = 0.072–0.113. D_1_: 0.161 × PLG + 19.633 × SPST/CS + 58.405 × GHL/CS − 19.08 < 0 (error 3.0% in 66 workers and 0.0% in 21 nest means). Color often homogeneously dark brown to blackish, with head only slightly lighter than gaster. So far only known from Crete. ………………………….……………………………………………………. ***ariadnae* sp. n.**

- Pubescence and hairs on 1^st^ gastral tergite longer: PLG/CS = 0.050–0.079, GHL/CS = 0.087–0.126. D_1_ > 0 (error 7.4% in 135 workers and 0.0% in 47 nest means). Color homogeneously brown to blackish, or heterogeneous with lighter reddish-brown head, mesosoma, and waist, and dark brown to blackish gaster. So far not known from Crete. ………………………………………………………………………………………. **2**

**2.**  Distances between frontal carinae and between spine tips smaller: FRS/CS = 347–387, SPTI/CS = 0.376–0.482. Mesosoma shorter: ML/CS = 1.010–1.104. Pubescence on 1^st^ gastral tergite longer: PLG/CS = 0.062–0.079. D_2_: 58.238 × FRS/CS + 23.193 × SPTI/CS − 145.744 × PLG/CS − 41.552 × POC/CL − 10.299 < 0 (error 0.0% in 29 workers). Color often homogeneously brownish, with head only slightly lighter than gaster. Aegean Islands and Anatolia. ……………………………………………………………………. ***ionia***

- Distances between frontal carinae and between spine tips larger: FRS/CS 360–435, SPTI/CS = 0.416–0.554. Mesosoma longer: ML/CS = 1.029–1.157. Pubescence on 1^st^ gastral tergite shorter: PLG/CS = 0.050–0.074. D_2_ > 0 (error 5.7% in 135 workers and 0.0% in 36 nest means). Color homogeneously brown to blackish, or heterogeneous with lighter reddish-brown head, mesosoma, and waist, and dark brown to blackish gaster. ……………………………………………………………………………………….… **3**

**3.**  Petiole and postpetiole narrower: PEW/CS = 0.329–0.384, PPW/CS = 0.269–0.320. Postocular distance smaller: POC/CL = 0.268–0.310. Scape longer: SL/CS = 0.771–0.845. D_3_ = 0.053 × SL + 77.406 × PLG/CS − 0.033 × SPST − 33.516 × FRS/CS − 0.055 × POC − 11.582 > 0 (error 5.0% in 40 workers and 0.0% in 13 nest means). Color homogeneously brown to blackish, or heterogeneous with lighter reddish-brown head, mesosoma, and waist, and dark brown to blackish gaster. Balkans south of 42° N. …………………………………………………………………………………. ***graeca* sp. n.**

- Petiole and postpetiole wider: PEW/CS = 0.345–0.430, PPW/CS = 0.277–0.329. Postocular distance larger: POC/CL = 0.280–0.336. Scape shorter: SL/CS = 0.750–0.837. D_3_ < 0 (error 3.0% in 66 workers and 0.0% in 23 nest means). Color often heterogeneous with lighter reddish-brown head, mesosoma, and waist, and dark brown to blackish gaster. Northwards until 47° N. ………………………………………………… ***schmidti***

### 3.5. Taxonomy of the East Mediterranean Crematogaster scutellaris Complex

***Crematogaster ariadnae* sp. n.**

ZooBank LSID: urn:lsid:zoobank.org:act:09733294-7739-4659-8FDA-C396B2E775E8

**Figure 8:**
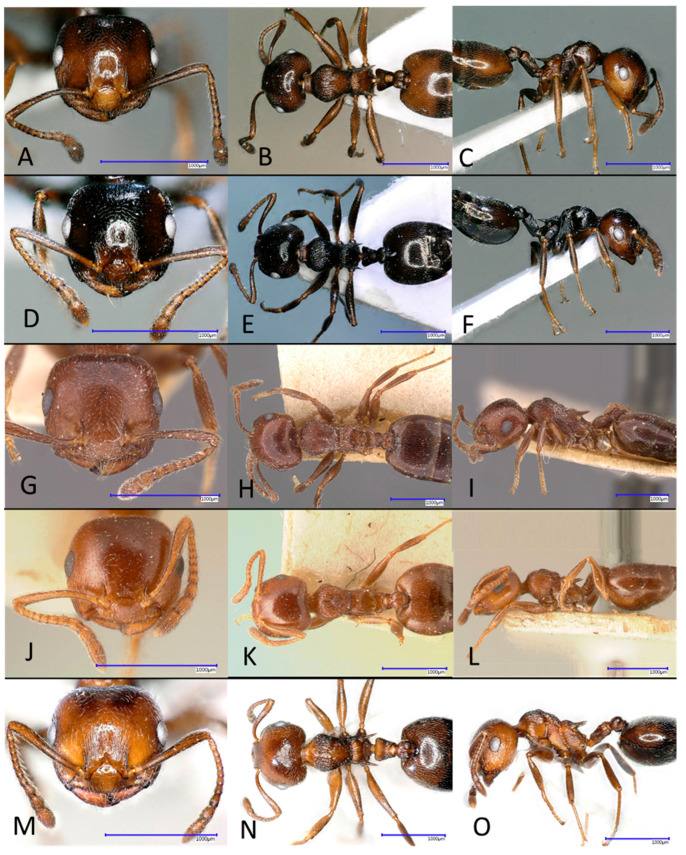
Holotype worker of *Crematogaster ariadnae* sp. n. in full-face (**A**), dorsal (**B**), and lateral (**C**) view (WAG3285); holotype worker of *Crematogaster graeca* sp. n. in full-face (**D**), dorsal (**E**), and lateral (**F**) view (WAG3254); worker of *C. graeca* sp. n. from Patras excluded from the type series of *Crematogaster ionia* Forel, 1911 in full-face (**G**), dorsal (**H**), and lateral (**I**) view (Photos: Z. Lieberman, AntWeb, CASENT0908491; consider that the dark color of this old specimen has faded over time); syntype worker of *Crematogaster ionia* Forel, 1911 from Izmir in full-face (**J**), dorsal (**K**), and lateral (**L**) view (Photos: C. Klingenberg, AntWeb, FOCOL1448; consider that the dark color of this old specimen has faded over time); dark worker of *Crematogaster schmidti* (Mayr, 1853) from Ankaran, Slovenia, in full-face (**M**), dorsal (**N**), and lateral (**O**) view (WAG3249).

**Figure 9:**
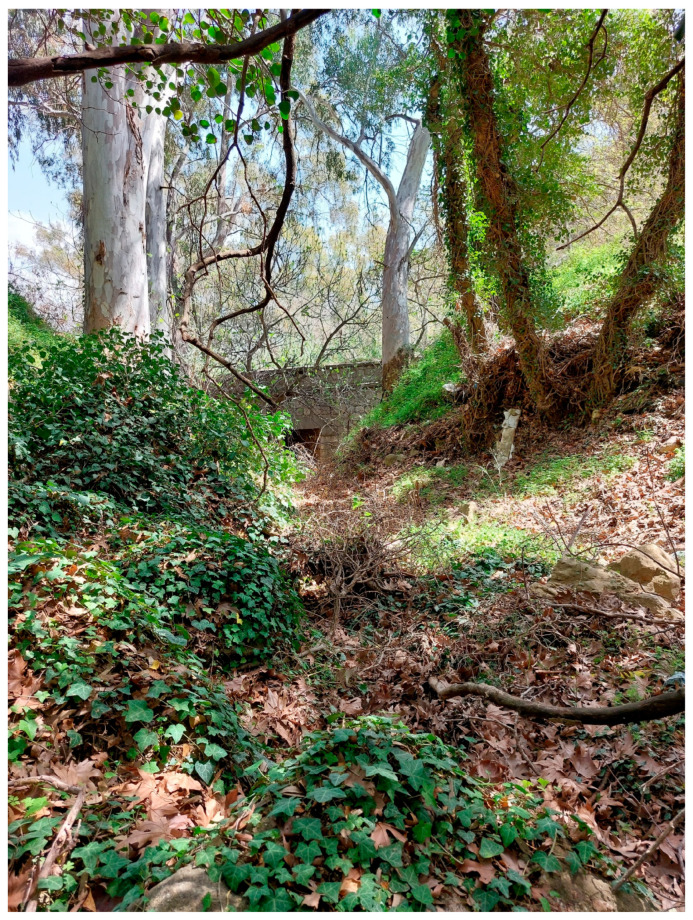
The type locality of *Crematogaster ariadnae* sp. n., a broadleaf wood along a swale with *Eucalyptus*, *Platanus orientalis*, *Hedera helix*, *Ficus carica*, *Rubus*, *Phragmites*, and *Olea europaea*.

**Holotype**. One worker (Figure 8), 0.8 km W Maronia, Crete (Greece), 35.1404° N, 26.0728° E, 180 m asl, broadleaf wood (with *Eucalyptus*, *Platanus orientalis*, *Ficus carica*, *Hedera helix*, *Rubus*, *Phragmites*, and *Olea europaea*) along a swale (Figure 9), 29.III.2024, H. C. Wagner legit, codename WAG3285 (upper specimen of its pin), NHMW, Austria.

**Paratypes.** Two workers from the holotype nest (WAG3285) and 12 mounted specimens from four other nests (WAG3276, WAG3282, WAG3287, WAG3291) from the same localities, deposited in NHMW, MHNG, SMNG, and SCPC.

**Etymology.** The specific epithet “*ariadnae*” refers to Ariadne from Greek mythology, who, on the island of Crete, provided Theseus a thread that allowed him to find his way through the labyrinth after slaying the Minotaur. The name alludes to the pronounced pheromone trails laid by this ant species, which are followed not only by nestmates but also by several camponotine ant species ([30]; *Camponotus gestroi*, *Ca. kiesenwetteri*, *Ca. lateralis*, *Ca. rebeccae*, and *Colobopsis imitans*), analogous to Ariadne’s guiding thread.

**Description of workers.**

Body color pattern: whole body often homogeneously dark brown to blackish, sometimes head, mesosoma, and first gastral tergite with a reddish component. Absolute cephalic size (CS) 869–1127 (mean = 971) µm. Cephalic length vs. maximum width of head capsule (CL/CW) 0.843–0.959 (mean = 0.905). Postocular distance vs. cephalic length (POC/CL) 0.278–0.316 (mean = 0.295). Postocular sides of cranium in frontal view convex. Vertex contour line in frontal view straight to concave. Vertex sculpture smooth, shiny, sometimes feebly areolate. Genae, in full-face view, anteriorly converging. Gena contour line feebly convex. Eye length vs. absolute cephalic size (EL/CS) 0.234–0.266 (mean = 0.253). Frontal carina distance vs. absolute cephalic size (FRS/CS) 0.362–0.415 (mean = 0.388). Median region of frons smooth, or feebly areolate, and shiny. Scape length vs. absolute cephalic size (SL/CS) 0.758–0.821 (mean = 0.787). Scape setae appressed to semierect with an angle of 0–15°. Spine length vs. absolute cephalic size (SPST/CS) 0.187–0.257 (mean = 0.213). Propodeal spine external width vs. absolute cephalic size (SPTI/CS) 0.358–0.528 (mean = 0.461). Maximum mesosoma width vs. absolute cephalic size (MW/CS) 0.579–0.653 (mean = 0.618). Dorsal region of pronotum: longitudinal costulate areolate ground sculpture, dull. Lateral region of pronotum: main sculpture forked costulate, rugulose with areolate ground sculpture. Dorsal region of propodeum: main sculpture forked costulate, rugulose with areolate ground sculpture. Mesopleuron and metapleuron areolate rugulose. Dorsal profile of petiolar node contour line in lateral view straight. Dorsal region of petiole sculpture: areolate ground sculpture, rarely superimposed by costulate-rugulose main sculpture. Dorsal region of postpetiole sculpture: areolate ground sculpture, rarely superimposed by costulate-rugulose main sculpture. Pubescence length on the 1^st^ gastral tergite (PLG/CS) 0.046–0.064 (mean = 0.054). The longest hair on the 1^st^ gastral tergite (GHL/CS) 0.072–0.113 (mean = 0.088).

**Diagnosis.**

This species is currently endemic to Crete. Available geographical data appear sufficient for successful separation. However, it remains uncertain whether *C. ariadnae* sp. n. occurs on neighboring islands or if other *C. scutellaris* complex species are present on Crete. In such cases, a simplified discriminant function based on three morphological characters (D_1_ = 0.161 × PLG + 19.633 × SPST/CS + 58.405 × GHL/CS − 19.08) can assist in telling apart *C. ariadnae* sp. n. samples from the other *C. scutellaris* complex species, achieving 100% classification success at the nest-series level.

D_1_ scores calculated for the nest-sample means are as follows:

*C. ariadnae* (*n* = 21) = −1.406 (−2.597, −0.087)

*C. graeca* (*n* = 13) = +1.419 (+0.274, +3.112)

*C. ionia* (*n* = 11) = +2.092 (+0.605, +3.760)

*C. schmidti* (*n* = 23) = +0.911 (+0.050, +2.111)

**Distribution.** Found at six localities on Crete: SW Lake Límni Agiás, 35.4747° N, 23.9316° E, 43 m, *Platanus* forest, 4.–5.IV.2024; 1.3 km E Souda, 35.4855° N, 24.0891° E, 2 m, *Eucalyptus* forest, 7.IV.2024; 1.4 km WSW Argiroupolis, 35.2822° N, 24.3191° E, 180 m, broadleaf wood along river (*Platanus orientalis*, *Olea europaea*, *Quercus coccifera*, *Ceratonia siliqua*, *Nerium oleander*), 2.–3.IV.2024; 1.6 km WNW Limenas Chersonisou, 35.3214° N, 25.3806° E, 25 m, humid *Eucalyptus*-palm forest, 31.III.–1.IV.2024; 0.5 km WNW Limne, 35.2494° N, 25.6336° E, 207 m, parking spot with pines, 31.III.2024; 0.8 km W Maronia, 35.1404° N, 26.0728° E, 222 m, humid *Eucalyptus*-*Platanus* swale with *Hedera helix*, 29.–30.III.2024.

**Ecology.** Nests on *Eucalyptus*, *Platanus orientalis*, *Pinus*, *Nerium oleander*, *Olea europaea*, *Quercus coccifera*, and *Ceratonia siliqua* (Figure 9). Trails were followed by *Camponotus gestroi*, *Ca*. *kiesenwetteri*, *Ca*. *lateralis*, *Ca. rebeccae*, and *Colobopsis imitans* [30].

***Crematogaster graeca* sp. n.**

ZooBank LSID: urn:lsid:zoobank.org:act:932CE7B8-CB76-483B-8C8A-6B6FB02F48DC

**Holotype.** One worker (Figure 8), W Piräus, near Athens (Greece), 37.9658° N, 23.558° E, 16 m asl, on *Pinus* in park, 25.III.2024, H. C. Wagner legit, codename WAG3254 (upper specimen of its pin), NHMW, Austria.

**Paratypes.** 11 workers from the holotype nest (WAG3254), deposited in NHMW, MHNG, SMNG, and SCPC.

**Etymology.** The specific epithet “*graeca*” refers to the nation of Greece, where most investigated material of this study is from and where the species is common.

**Description of workers.**

Body color pattern: head, mesosoma, and waist homogeneous dark brown to reddish; gaster dark brown to blackish. Absolute cephalic size (CS) 941–1160 (mean = 1034) µm. Cephalic length vs. maximum width of head capsule (CL/CW) 0.864–0.963 (mean = 0.898). Postocular distance vs. cephalic length (POC/CL) 0.268–0.310 (mean = 0.287). Postocular sides of cranium in frontal view convex. Vertex contour line in frontal view straight to concave. Vertex sculpture smooth, shiny. Genae, in full-face view, anteriorly converging. Gena contour line feebly convex. Eye length vs. absolute cephalic size (EL/CS) 0.235–0.273 (mean = 0.255). Frontal carina distance vs. absolute cephalic size (FRS/CS) 0.360–0.396 (mean = 0.380). Median region of frons smooth, or feebly areolate, shiny. Scape length vs. absolute cephalic size (SL/CS) 0.771–0.845 (mean = 0.800). Scape setae appressed to semierect with an angle of 0-15°. Spine length vs. absolute cephalic size (SPST/CS) 0.182–0.244 (mean = 0.217). Propodeal spine external width vs. absolute cephalic size (SPTI/CS) 0.416–0.531 (mean = 0.467). Maximum mesosoma width vs. absolute cephalic size (MW/CS) 0.594–0.651 (mean = 0.622). Dorsal region of pronotum: longitudinal costulate areolate ground sculpture, dull. Lateral region of pronotum: main sculpture forked costulate, rugulose with areolate ground sculpture. Dorsal region of propodeum: main sculpture forked costulate, rugulose with areolate ground sculpture. Mesopleuron and metapleuron areolate rugulose. Dorsal profile of petiolar node contour line in lateral view straight to feebly concave. Dorsal region of petiole sculpture: areolate ground sculpture, rarely superimposed by costulate-rugulose main sculpture. Dorsal region of postpetiole sculpture: areolate ground sculpture, rarely superimposed by costulate-rugulose main sculpture. Pubescence length on the 1^st^ gastral tergite (PLG/CS) 0.050–0.074 (mean = 0.063). The longest hair on the 1^st^ gastral tergite (GHL/CS) 0.087–0.126 (mean = 0.100).

**Diagnosis**

Since coloration overlaps with other species, we propose a discriminant function based on eight morphometric characters (D_4_ = 0.051 × PLG + 0.031 × SL + 0.007 × SPTI + 35.216 × EL/CS + 0.013 × ML − 0.016 × SPST − 0.056 × POC − 0.025 × PEW − 26.479) for separating *C. ionia* from all other species treated in this study, yielding classification success without overlap at the nest-series level.

*C. graeca* sp. n. (*n* = 13) = +1.071 (+0.169, +2.056)

*C. ariadnae* sp. n. (*n* = 21) = −1.798 (−3.374, −0.196)

*C. ionia* (*n* = 11) = −1.256 (−2.384, −0.128)

*C. schmidti* (*n* = 23) = −1.838 (−3.242, −0.185)

**Distribution.** Found on eight localities in Central Greece, Attica, the Peloponnese, and North Macedonia: Patras [38.25° N, 21.73° E], IV.-V.1910, leg. A. Forel, CASENT0908491 [from AntWeb], one syntype worker of *C. ionia*; Star Dojran center, 41.1798° N, 22.7254° E, 154 m, park, 15. and 18.IV.2010; 0.7 km S Star Dojran, 41.1735° N, 22.7259° E, 212 m, bushland, 20.IV.2010; 4.3 km ENE Astros, 37.4212° N, 22.7666° E, 10 m, *Pinus* forest, 8.IV.2024; Arktisa, 38.7455° N, 23.0416° E, 2 m, *Eucalyptus* tree in meadow, 24.III.2024; 1.2 km N Palea Epidauros, 37.6492° N, 23.1543° E, 143 m, *Pinus* forest, 9.IV.2024; 2.2 km NNE Palea Epidauros, 37.6582° N, 23.1631° E, 8 m, *Pinus* forest, 9-10.IV.2024; W Piräus, 37.9658° N, 23.558° E, 16 m, *Pinus* in park, 25.III.2024.

**Ecology.** Nests on *Eucalyptus*, *Pinus*, *Olea europaea*, *Cupressus*, and in a ruin wall. Trails were followed by *Camponotus gestroi*, *Ca*. *kiesenwetteri*, and *Ca*. *lateralis* [30].

***Crematogaster ionia* Forel, 1911**

*Crematogaster scutellaris* var. *ionia* Forel, 1911: 340; raised to species rank: Agosti and Collingwood, 1987: 54. Syntype morphology investigation hereby.

**Type localities.** The specimens comprising the original type series were collected from widely separated localities: “Boudja et Coccarinali, près Smyrne; presqu’île d’Aivaly, près Mitylène (toujours dans le tas de bois d’Aivaly, importé à Smyrne); Ismid (Bithynia); Kephirissa, près d’Athènes; Patras; Corfou (Grèce)]” [56]. These localities fall within the known distribution range of two parapatric species (*C. graeca* sp. n. and *C. ionia*), raising the possibility that representatives of both species are intermixed within the type series. We suggest giving precedence to the Anatolian taxon over the Greek mainland one, as it occurs within the historical region of Ionia (i.e., the west coast of Anatolia), from which the species epithet is derived.

A syntype worker is labeled “’Smyrna [Izmir] / Forel.’ [–] ‘Crematogaster / scutellaris Ol. / ionia For.’ [–] Typus [–] Forel ded. [sic!] 1922”. It is housed in the ZMB, pictures on AntWeb have the ID FOCOL1448 (Figure 8). In an LDA wild-card run, the syntype worker of *C. ionia* was assigned with 0.711 to the cluster with material from Samos, Karpathos, and Rhodes.

We exclude a syntype worker of *Crematogaster ionia* from the type series, it is labeled “’Cr. scutellaris / Ol. / v. ionia Forel. / Patras, Grece [sic!] / (Forel)’ [–] Typus [–] Coll. / A. Forel [–] ANTWEB / CASENT / 0908491”. In an LDA wild-card run, the type worker was assigned to the cluster from Greek mainland, which we describe here as *C. graeca* sp. n., with 0.990 [digitally measured from the AntWeb images].

**Redescription of workers.**

Body color pattern: head, mesosoma, and waist homogeneous dark brown, sometimes with a reddish component; gaster dark brown to blackish. Absolute cephalic size (CS) 891–1071 (mean = 993) µm. Cephalic length vs. maximum width of head capsule (CL/CW) 0.884–0.923 (mean = 0.900). Postocular distance vs. cephalic length (POC/CL) 0.280–0.324 (mean = 0.301). Postocular sides of cranium in frontal view convex. Vertex contour line in frontal view straight to concave. Vertex sculpture smooth, shiny, sometimes feebly areolate. Genae, in full-face view, anteriorly converging. Gena contour line feebly convex. Eye length vs. absolute cephalic size (EL/CS) 0.236–0.258 (mean = 0.247). Frontal carina distance vs. absolute cephalic size (FRS/CS) 0.347–0.387 (mean = 0.366). Median region of frons smooth, or feebly areolate, shiny. Scape length vs. absolute cephalic size (SL/CS) 0.759–0.813 (mean = 0.788). Scape setae appressed to semierect with an angle of 0–30°. Spine length vs. absolute cephalic size (SPST/CS) 0.176–0.241 (mean = 0.205). Propodeal spine external width vs. absolute cephalic size (SPTI/CS) 0.376–0.482 (mean = 0.432). Maximum mesosoma width vs. absolute cephalic size (MW/CS) 0.572–0.676 (mean = 0.610). Dorsal region of pronotum: longitudinal costulate areolate ground sculpture, dull. Lateral region of pronotum: main sculpture forked costulate, rugulose with areolate ground sculpture. Dorsal region of propodeum: main sculpture forked costulate, rugulose with areolate ground sculpture. Mesopleuron and metapleuron areolate rugulose. Dorsal profile of petiolar node contour line in lateral view straight, feebly concave. Dorsal region of petiole sculpture: areolate ground sculpture, rarely superimposed by costulate-rugulose main sculpture. Dorsal region of postpetiole sculpture: areolate ground sculpture, rarely superimposed by costulate-rugulose main sculpture. Pubescence length on the 1^st^ gastral tergite (PLG/CS) 0.062–0.079 (mean = 0.070). The longest hair on the 1^st^ gastral tergite (GHL/CS) 0.087–0.121 (mean = 0.105).

**Diagnosis.**

This species is readily distinguished from *C. schmidti* by coloration: the latter is frequently bicolorous, with a reddish mesosoma and a reddish head, whereas the former is rather uniformly dark throughout.

**Figure 10:**
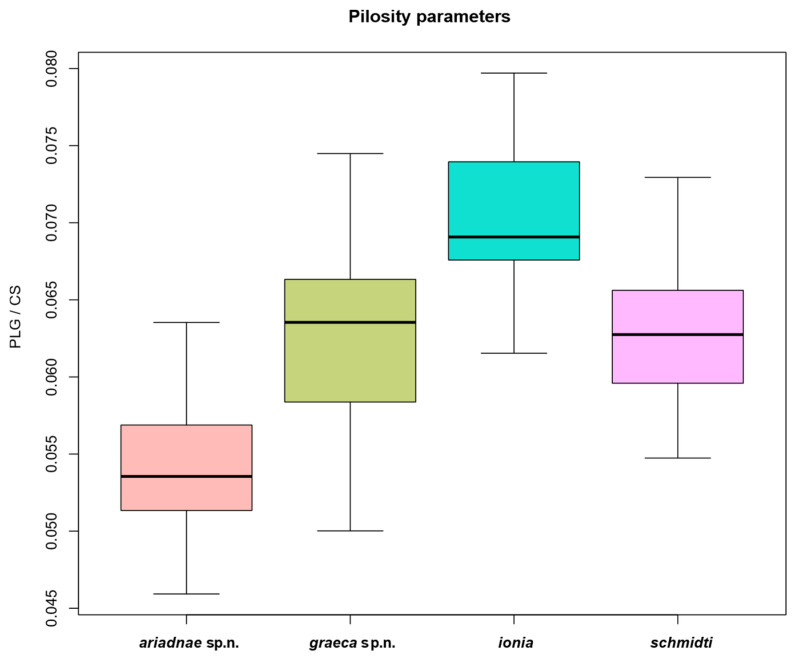
Boxplot illustrating distribution of gaster pilosity parameters (PLG/CS) at the individual level for four studied species.

*Crematogaster ionia* closely resembles *C. ariadnae* sp. n. and *C. graeca* sp. n. in overall appearance, sharing dark brown coloration and a similar sculpture with both species. It is, however, distinguished by possessing the highest gaster-pubescence pilosity index (PLG/CS) within the species complex (0.070 ± 0.002 (95% confidence intervall)), in contrast to the other two often dark-colored taxa, in which PLG/CS values are considerably lower (0.063 ± 0.002 and 0.054 ± 0.001, respectively; Table 4, Figure 10). In ambiguous or otherwise problematic cases, a discriminant function incorporating four morphometric characters (D_5_ = 0.12 × PLG + 0.041 × POC + 0.012 × CS − 0.055 × FRS − 0.021 × SPTI − 1.209) may serve as a useful diagnostic aid, achieving 100% classification success at the nest-series level.

*C. ionia* (*n* = 11) = +1.696 (+0.761, +2.623)

*C. ariadnae* (*n* = 21) = −2.279 (−3.241, −1.083)

*C. graeca* (*n* = 13) = −1.202 (−2.452, −0.278)

*C. schmidti* (*n* = 23) = −1.373 (−3.244, −1.183)

**Distribution.** Found at seven localities on Samos, Karpathos, Rhodes, and in Anatolia: Drakei, 37.769° N, 26.633° E, 29 m, broadleaf forest, 8.VI.2013; Nachtigallental, 37.7833° N, 26.8167° E, 500 m, broadleaf forest, 9.VI.2013; Pythagorio, 37.683° N, 26.933° E, 20 m, 5.VI.2013; Psili Ammos, 37.7° N, 27.0° E, 10 m, 4.VI.2013; 5.7 km N Spoa, 35.6877° N, 27.1504° E, 192 m, *Pinus* forest, 26.-27.III.2024; Smyrna [Izmir, 38.38° N, 27.17° E], IV.-V.1910, leg. A. Forel, FOCOL1448; 1.2 km NW Kiotari, 36.0556° N, 27.9472° E, 65 m, 2.VII.2008, leg. L. Borowiec, CASENT0193617 [from AntWeb].

**Ecology.** Nests on *Pinus* and broadleaf trees. Trails were followed by *Camponotus lateralis* and *Ca. kiesenwetteri* [30].

***Crematogaster schmidti* (Mayr, 1853)**

*Acrocoelia schmidti* Mayr, 1853: 149; combination in *Crematogaster*: Mayr, 1855: 469; junior synonym of *Crematogaster scutellaris*: Mayr, 1855: 469; raised to species rank: Emery, 1891: 14; ranked as subspecies of *Crematogaster scutellaris*: Forel 1904: 372; raised to species rank: Collingwood, 1961: 64; junior synonym of *Crematogaster scutellaris*: Fromantin and Soulié, 1961: 95; ranked as species: Arnol’di and Dlussky, 1978: 538.

*Crematogaster scutellaris christowitchii* Forel, 1892; subspecies of *Crematogaster scutellaris*: Dalla Torre 1893: 86; junior synonym of *Crematogaster schmidti*: Emery 1922: 143; synonymy confirmed by morphometric analyses hereby.

*Crematogaster auberti* subsp. *karawaewi* Ruzsky, 1905: 497; junior synonym of *Crematogaster schmidti*: Dlussky et al., 1990: 244; synonymy confirmed based on zoogeography and original description hereby.

*Crematogaster scutellaris medispina* Forel, 1905; junior synonym of *Crematogaster schmidti*: Emery, 1922; synonymy confirmed by morphometric analyses hereby.

**Type locality.** Wipbacher Thale [Vipava Valley] in Krain (Slovenia) [45.848° N, 13.963° E].

**Type material.** The type worker is labeled “’zu G. Mayr / Bd. II. p. 143’ [–] ‘Krain’ / Coll. G. Mayr [–] ANTWEB / CASENT / 0919655 [–] Type [–] ‘Acroc. / schmidti Mayr.’ / det. G. Mayr”. It is housed in the NHMW, pictures on AntWeb have the ID CASENT0919655. This type was not available for morphometric investigation because of damage, but color and zoogeography strongly match with *C. schmidti*.

A further worker from Dalmatia is labeled as “’Cr. Schmitti [sic!] / ☿ Mayr / Donné par / Mayr, d’après / lui absol. / identique / au type’ [–] Cotypus [–] Dalmatien / Ragusa [Dubrovnik] / Reitter. [–] Coll. / A. Forel [–] ANTWEB / CASENT / 0908487”. It is housed at the MHNG. In an LDA wild-card run, this cotype worker was assigned to *C. schmidti* with 0.945 [digitally measured from the AntWeb images].

Two syntype workers of *Crematogaster scutellaris christowitchii* Forel, 1892 are labeled “C. scutellaris Ol / v. christovichii [sic!] / Forel / Tatar Bazarnik [Pazardzhik] [–] TYPUS [–] MHNG-ENTO-0296684” and “C. scutellaris Ol / v. christovichii [sic!] / Forel / Sliven / Bulgarie [–] TYPUS [–] MHNG-ENTO-0296687”. They are housed in the MHNG. In an LDA wild-card run, these type workers were assigned to *C. schmidti* with 0.620 and 0.898. The combination of its zoogeographic origin [Bulgaria] and information provided in the original description (“Roth, mit schwarzem Hinterleib”) affirms the conspecificity with *C. schmidti* (cf. [47]).

*Crematogaster auberti* subsp. *karawaewi* Ruzsky, 1905 is considered a junior synonym of *C. schmidti*. The combination of its zoogeographic origin [Crimea] and information in the original description (“The spines on the propodeum are of medium size (they are shorter than those of *auberti* and longer than those of *laestrygon*) … The overall coloration is reddish-brown, with a blackish-brown abdomen (except for a small brownish spot at its base) and a dark upper surface of the head” [in Russian]) [77] fits best with *C. schmidti* (cf. [21]). Due to the Ukrainian war, type material is not available currently.

A worker of *Crematogaster scutellaris medispina* Forel, 1905 is labeled “’v. medispina / Forel / Type’ [–] ‘Cr. scutellaris / ☿ Ol.’ [–] Typus [–] ‘v. medispina / For’ [–] Adrianopel [Edirne] / V194 Flach. [–] Coll. / A. Forel [–] ANTWEB / CASENT / 0908488”. It is housed at the MHNG, pictures on AntWeb have the ID CASENT0908488. In an LDA wild-card run, this type worker was assigned to *C. schmidti* with 0.984 [digitally measured from the AntWeb images].

**Redescription of workers.**

Body color pattern: head, mesosoma, and waist typically reddish; gaster dark brown to blackish, first gastral tergite sometimes with a reddish component. In the northwestern part of its area, sometimes with a blackish mesosoma and waist as a consequence of introgression from *C. scutellaris* [17,18]. Absolute cephalic size (CS) 816–1136 (mean = 974) µm. Cephalic length vs. maximum width of head capsule (CL/CW) 0.871–0.932 (mean = 0.902). Postocular distance vs. cephalic length (POC/CL) 0.280–0.336 (mean = 0.298). Postocular sides of cranium in frontal view convex. Vertex contour line in frontal view straight to concave. Vertex sculpture feebly costulate, ground sculpture smooth to feebly areolate. Genae, in full-face view, anteriorly converging. Gena contour line feebly convex. Eye length vs. absolute cephalic size (EL/CS) 0.241–0.271 (mean = 0.254). Frontal carina distance vs. absolute cephalic size (FRS/CS) 0.364–0.435 (mean = 0.387). Median region of frons feebly costulate, ground sculpture smooth to feebly areolate. Scape length vs. absolute cephalic size (SL/CS) 0.750–0.837 (mean = 0.784). Scape setae appressed to semierect with an angle of 0-15°. Spine length vs. absolute cephalic size (SPST/CS) 0.197–0.251 (mean = 0.221). Propodeal spine external width vs. absolute cephalic size (SPTI/CS) 0.426–0.554 (mean = 0.478). Maximum mesosoma width vs. absolute cephalic size (MW/CS) 0.595–0.663 (mean = 0.624). Dorsal region of pronotum: longitudinal costulate areolate ground sculpture, dull. Lateral region of pronotum: main sculpture forked costulate, rugulose with areolate ground sculpture. Dorsal region of propodeum: main sculpture forked costulate, rugulose with areolate ground sculpture. Mesopleuron and metapleuron areolate rugulose. Dorsal profile of petiolar node contour line in lateral view straight, feebly concave. Dorsal region of petiole sculpture: areolate ground sculpture, rarely superimposed by costulate-rugulose main sculpture. Dorsal region of postpetiole sculpture: areolate ground sculpture, rarely superimposed by costulate-rugulose main sculpture. Pubescence length on the 1^st^ gastral tergite (PLG/CS) 0.055–0.073 (mean = 0.063). The longest hair on the 1^st^ gastral tergite (GHL/CS) 0.090–0.112 (mean = 0.100).

**Diagnosis.**

*Crematogaster schmidti* exhibits usually a reddish head, mesosoma, and waist with dark brown to black gaster. The other three species are often homogeneously dark colored, a safe discrimination from the three other species can be realized using the three discriminants given above.

**Distribution.** Found at eleven localities on the Balkans: 2.9 km NW Ankaran, 45.5913° N, 13.7042° E, 17 m, *Pinus* forest, 15.X.2013; 1.6 km NW Ankaran, 45.5908° N, 13.7234° E, 18 m, *Quercus*-*Ulmus* forest with *Hedera helix*, 17.-19.IV.2023 and 14.-15.X.2023; Wipbacher Thale [Vipava Valley] in Krain (Slovenia) [45.848° N, 13.963° E], leg. F. Schmidt, CASENT0919655 [from AntWeb]; 9.5 km W Orebić, 42.9851° N, 17.0676° E, 27 m, macchia, 4.-5.IV.2023; Dubrovnik, [42.65° N, 18.09° E], leg. Reitter, CASENT0908487 [from AntWeb]; 3.8 km SE Petrovac, 42.1836° N, 18.9759° E, 5 m, on *Quercus* trees, 2.-3.IV.2023; 2.7 km SSE Ladochori, 39.4685° N, 20.2476° E, 9 m, broadleaf forest (*Quercus coccifera*, *Olea europaea*, *Pistacia lentiscus*), 15.-16.IV.2024; 2.0 km SW Araxos, 38.1755° N, 21.3683° E, 2 m, *Quercus* forest, 12.-13.IV.2024; Tatar-Bazarnik [Pazardzhik, 42.19° N, 24.33° E], VIII.1891, leg. A. Forel; Sliven [42.68° N, 26.32° E], VIII.1891, leg. A. Forel; Adrianopel [Edirne, 41.68° N, 26.56° E], leg. D. Flach CASENT0908488 [from AntWeb].

**Ecology.** Nests on *Quercus ilex*, *Q. coccifera*, *Q. ithaburensis*, *Q. pubescens*, *Pinus* spp., *Olea europaea*, *Pistacia lentiscus*, *Ulmus*, *Prunus*, *Acer campestris*, in unidentified shrubs, and in a ruin. Trails were followed by *Camponotus lateralis*, *Ca. dalmaticus*, *Ca. gestroi*, *Colobopsis truncata*, and *Co. imitans* [30,38]. Probably less thermophilic than the three other species.

## 4. Discussion

The present study suggests the presence of four east Mediterranean species of the *Crematogaster scutellaris* complex: *C. ariadnae* sp. n., *C. graeca* sp. n., *C. ionia*, and *C. schmidti*. Based on morphometric similarity, *C. ionia* might be phylogenetically most distant, while *C. ariadnae* sp. n. is the sister species of *C. schmidti*.

A limitation of our study is the incomplete geographic coverage, particularly for *C. schmidti* and *C. ionia*. Sampling of *C. schmidti* in the present dataset was restricted to the Balkans, although this species has also been reported from Anatolia [52,53], Crimea [21], the Caucasus [54,78], and Iran [55]. In *C. ionia*, no material from populations east of Rhodes was morphometrically investigated, although the species has also been reported from Cyprus [59] and the Levant [38,79,80]. Consequently, geographically structured variation within these taxa may still be insufficiently represented. Nevertheless, the four-species hypothesis is supported by consistent morphometric separation and low classification error rates in the LDA. Additional material from currently unsampled regions, especially Cyprus and the Levant, will be important for testing the stability of the proposed diagnostic characters and the taxonomic arrangement in future research.

The four species considered in this region show a clear zoogeographic pattern: *Crematogaster schmidti* is widespread on the Balkans and goes farthest to the north, *C. graeca* sp. n. was found only in the Greek mainland and North Macedonia. *Crematogaster ionia* occurs on the Eastern Aegean islands and in Western Anatolia; a wider distribution in Anatolia is zoogeographically expected. Future research should uncover the transition zone of *C. graeca* sp. n. and *C. ionia* s. str. in Bulgaria, Turkish Thrace, and Northwest Anatolia, where *C. ionia* s. l. is also known [47,60,81,82]. Finally, we expect that *C. ariadnae* sp. n. is endemic to Crete.

It is an interesting fact that the species of the *Crematogaster scutellaris* complex, which are highly aggressive and territorial [17,30], tend to ecologically exclude one another. We have already observed this pattern in the distribution of *C. scutellaris* and *C. schmidti* in the Northwest Balkans [30] and do not know any convincing case in which more than one species of the *C. scutellaris* complex were detected at one locality. No ecological or phenotypic differences between the species of the *C. scutellaris* complex are known, and the taxa appear to occupy similar thermophilous woodland habitats [10,17]. Based on the more northern distribution of *C. schmidti*, we suspect a lower thermophily than in *C. ariadnae* sp. n., *C. graeca* sp. n., and *C. ionia*. Concerning the speciation of the latter three, the separation of their ranges by the deep waters of the Aegean Sea over millions of years suggests speciation through geographic isolation: *C. ariadnae* sp. n. on Crete, *C. graeca* sp. n. on the southernmost area of the Balkan mainland, and *C. ionia* in Anatolia. Similarly, the speciation of *C. scutellaris* and *C. schmidti* was probably also initiated by separation into a western and an eastern refugium [17].

## Figures and Tables

**Table 1:** Verbatim definitions of morphometric characters.

Abbr.	Verbal Character Definition
**CL**	Maximum cephalic length in median line; head must be carefully tilted to position with actual maximum. Excavations of hind vertex and/or clypeus, if any, reduce CL (Figure 1A).
**CS**	Cephalic size: arithmetic mean of CL and CW.
**CW**	Maximum width of head capsule, measured across eyes (Figure 1A).
**EL**	Maximum diameter of a compound eye (Figure 1B).
**FRS**	Minimum distance between frontal carinae (Figure 1A).
**GHL**	Longest erect hair on 1^st^ gastral tergite, typically found near posterior border of tergite.
**ML**	Mesosoma length from caudalmost point of propodeal lobe to transition point (=point of inflection) between convex anterior pronotal slope and concave anterior pronotal shield in lateral view (Figure 1B).
**MW**	Maximum pronotal width (Figure 1C).
**NOL**	Length of petiolar node, measured in lateral view from petiolar spiracle to the nearest point of the caudal cylinder (Figure 1B).
**PEW**	Maximum width of petiole (Figure 1C).
**PLG**	Arithmetic mean of three longest pubescence hairs on central area of 1^st^ gastral tergite.
**POC**	Postocular distance. Use cross-scaled ocular micrometer and adjust head to CL measuring position. Caudal measuring point: median occipital margin; frontal measuring point: median head at level of posterior eye margin (Figure 1A).
**PPW**	Maximum width of postpetiole (Figure 1C).
**SL**	Maximum straight line scape length excluding articular condyle (Figure 1A).
**SPST**	Distance between center of propodeal stigma and spine tip. The stigma center refers to midpoint defined by outer cuticular ring, but not to center of real stigma opening that may be positioned eccentrically (Figure 1B).
**SPTI**	Distance between spine tips in dorsal view (Figure 1C).

**Table 2:** The assignment probabilities of the investigated type material of the four species of the *Crematogaster scutellaris* complex obtained by a wild-card LDA.

Type	*ariadnae* sp. n.	*graeca* sp. n.	*ionia*	*schmidti*
*schmidti* paratype CASENT0908487	0.031	0.001	0.023	**0.945**
*medispina_*syntype CASENT0908488	0.002	0.013	0.001	**0.984**
*christowitchii* MHNG-ENTO-0296684	0.379	0.001	<0.001	0.620
*christowitchii* MHNG-ENTO-0296687	0.088	0.014	<0.001	0.898
*ionia* syntype FOCOL1448	0.011	0.130	0.711	0.148
*ionia* syntype CASENT0908491	0.001	**0.990**	0.001	0.008

Values > 0.9 in bold.

**Table 3:** Worker-individual error rates of cross-validation LDAs for pairwise species comparisons [%] with the number of characters used in parenthesis.

	*ariadnae* sp. n.	*graeca* sp. n.	*ionia*	*schmidti*
*n*/*i*	21/66	13/40	11/29	23/66
*ariadnae* sp. n.				
*graeca* sp. n.	**1.9** (5)			
*ionia*	**0.0** (2)	5.8 (4)		
*schmidti*	**3.0** (7)	**4.7** (5)	**0.0** (8)	

*n* = number of nests, *i* = number of individuals. Values < 5% in bold.

## Data Availability

The original contributions presented in this study are included in the article/Supplementary Materials. Further inquiries can be directed to the corresponding author.

## Supplementary Materials

The following supporting information can be downloaded at: https://www.mdpi.com/article/10.3390/insects17060658/s1, Table S1: morphometric data.
